# Molecular Imprinted Silica with West Nile Antibody Templates show Specific and Selective Binding in Immunoassays

**DOI:** 10.4172/2155-952x.1000260

**Published:** 2017-06-13

**Authors:** Julio E Rincon, Fabio Diaz Santillan, Pedro M Palermo Infante, Douglas M Watts, Thomas Boland

**Affiliations:** 1 Biomedical Engineering, University of Texas at El Paso, El Paso, TX, USA; 2 Biological Sciences, University of Texas at El Paso, El Paso, TX, USA

**Keywords:** Molecularly imprinted polymer particles (MIPs), Immunoassay test, Charge matched molecular imprinting

## Abstract

A new molecular imprinting technique was developed for molecularly imprinted polymer particles (MIPs). Particles were synthesized using organic silane chemistries by a sol-gel process, where the relative amount of active monomers was complementary matched to the relative amount of surface charges of the West Nile antibody template. Synthesized MIPs showed specific binding to affinity purified polyclonal West Nile antibodies (WNA) with a loading capacity of 80 µg/mg, while MIPs absorbed non-specific proteins at a loading capacity of 28 µg/mg. A dissociation constant of Kd=57.45 μM was measured from the binding isotherms. MIPs selectively absorbed 27 times more WNA than either albumin or immunoglobulin, while MIPs absorbed 16 times more WNA than non- imprinted particles (NIPs). Finally, fluorescently labeled MIPs were incubated in a high bind 96 well plate previously loaded with template, albumin, or immunoglobulin as an immunoassay test. Fluorescent MIPs significantly bound more to wells with WNA than any other control. Thus, the development of new affordable and robust immunoassays with MIPs would be possible in the future.

## Introduction

There is a significant demand for robust and stable receptor molecules that can mimic biological molecules, such as antibodies [1]. Relying only on natural recognition molecules has limited the uses and capabilities of many aspects of health sciences due to product expense and stability. These limitations have impacted low resource areas where product expense and limited cold-chain makes antibody-based diagnostics tough to implement. The absence of diagnostic tests limits disease treatment based their clinical symptoms and the local prevalence of the disease. While this method is generally effective, unnecessary or inadequate treatment may be administered.

In low-resource settings, lateral flow assays are used because of their speed, simplicity, and relatively low cost. Unfortunately, these assays have poor sensitivity to many analytes and have the inability to multiplex [2,3]. Furthermore, the antibodies used in these devices suffer from product stability, slow and expensive manufacturing, and temperature requirements can be difficult to maintain [4,5]. As an alternative, molecular imprinting has been proposed to fabricate robust immunoassays [6–8].

Molecular imprinting is a chemical process that generates particles with artificial recognition sites, which can specifically bind target molecules similarly to antibodies. Molecularly imprinted polymer particles (MIPs) require monomers to self-assemble around the presence of a template. As monomers conform to the shape of the template, they are fixed by a rapid polymerization of the network, thus forming MIPs. Subsequent removal of the template leaves active sites in MIPs, capable of specifically recognizing the original template [9–11]. MIPs have the advantage of being stable at any temperature, can be stored dry for several years, and can be rapidly manufactured at any scale [12].

However, MIPs suffer from difficulties imprinting molecules larger than 1,500 Da [13–16], while the majority of immunogenic antigens are typically greater than 6,000 Da. Limitations of molecular imprinting are due to multiple factors such as size, complexity and conformational structure of the protein template [17–19]. Moreover, the majority of imprinting technologies require organic solvents [19] where a protein template will denature before an imprint can be formed; organic solvents are necessary to increase short-range interactions between the template and MIPs.

While developing MIPs compatible with aqueous environments are of great importance, this is difficult to achieve. Short range MIPs- antigen interactions are reduced due to competition between ions and water molecules in aqueous environments; hence a strong bind with the template could be prevented [20]. However, natural antibodies are capable of displacing hydration layers with long range interactions, thus establishing intimate contact with the antigen. It is then imperative to customize active monomers type and quantity depending on the template used.

After rendering West Nile antibodies (WNA), using available crystallographic data, molecular imprinting was achieved. MIPs active monomers were complementary matched to positive and negative amino acids in WNA. Hydrophobic amino acids were matched at an experimentally determined ratio, as not all hydrophobic amino acids are exposed in the template. We used the following active monomers: 3-aminopropyl triethoxysilane (APS), as the positive monomer; carboxybutyl 3-Amidepropyl triethoxysilane (cAPS), as the negative monomer; octyl triethoxy silane, as the hydrophobic monomer. As the backbone monomer, we used tetraethyl orthosilicate (TEOS).

## Materials and Methods

Tetraethyl orthosilicate (TEOS), 3-aminopropyl triethoxysilane (APS), phosphate buffered saline (PBS), succinic anhydride, ammonium hydroxide, sodium hydroxide, and ATTO 495 NHS ester were purchased from Sigma-Aldrich, all reagent grade. Octyl triethoxysilane (OTS) was purchased from Sigma-Aldrich at 95% purity. Hydrochloric acid (37%), anhydrous acetic acid, and ethanol were purchased from Fisher Scientific, reagent grade. Bovine serum albumin (BSA) and bovine gamma globulin (BGG) standard ampules were purchased from Thermo Scientific. 4-(2-Hydroxyethyl)piperazin-1-ylethanesulphonic acid (HEPES) was purchased from VWR, reagent grade.

Polymer grafted carbon black was obtained by collecting ink from HP 33 cartridges (HP, Palo Alto, CA). Ultrapure water was obtained from a Milli-Q Millipore unit with a water quality of 18.2 MΩ. Elution buffer was made in DI water with 0.15 M NaCl and 0.5% acetic acid with a pH of 3.5. HEPES buffer was prepared with 25 mM of HEPES and 125 mM of NaCl at a pH 7.4 in DI water. Thermo Scientific Orion 3 Star and 2 Star benchtop pH meters were used to determine pH in the buffers and MIPs vials respectively. Biomate 3 UV-Vis spectrophotometer was used for protein determination with BCA and micro BCA kits from Fisher Scientific.

### Preparation of cAPS

Carboxybutyl 3-Amidepropyl triethoxysilane (cAPS) was synthesized by reacting 5 ml of APS with 2.137 g of succinic anhydride and adding ethanol to a final volume of 15 ml; the solution was then left in a rotisserie overnight. The solution was then centrifuged for five minutes at 3200 g to separate unreacted succinic anhydride from the supernatant. Afterward, the presence of carboxyl groups was confirmed with a Thermo Nicolet Nexus FTIR.

### Preparation of West Nile antibodies

WNA were obtained from hyperimmune mouse ascitic fluid (HMAF) and were prepared as described in article [21]. WNA antibodies were then purified by affinity gel goat antibody to mouse IgG column from MP Biomedicals (Santa Ana, CA). Briefly, the affinity column was equilibrated with a 2x PBS buffer solution. Afterward, 1.0 ml of serum- containing WNA was added. The affinity column was then rinsed with 10 ml of PBS. Antibody was recuperated from the column by flowing 5 ml of elution buffer; eluent was collected, and protein concentration was determined with UV-VIS at 280 nm. Eluted sample was then desalted with a Hitrap 5 ml desalting column (General Electric Healthcare Life Sciences, Wauwatosa, WI) previously equilibrated with HEPES buffer at a pH of 7.4.

### Preparation of fluorescently labeled proteins

For confocal imaging, WNA, BSA, and BGG were dyed using ATTO 495 NHS ester at a protein concentration of 120 µg/ml. Briefly, each protein was desalted in separate Hitrap desalting columns, previously equilibrated in 0.1 M bicarbonate buffer at a pH of 8.3. Afterward, 75 µl of ATTO 495 NHS was added to 1.5 ml of the respective protein. The solution was incubated for one hour at room temperature. Afterward, Hitrap columns were equilibrated with 1x PBS and the proteins were desalted. We obtained proteins at an average concentration of 100 µg/ml.

### Preparation of molecularly imprinted particles

Imprinted silica particles were prepared by a modified sol-gel method as described in [22–27]. First, we obtained the crystallographic data of a West Nile Fab antibody (database identifier: 3N9G) from the NIH molecular modeling database (MMDB). We then determined the active monomers and their volumes by complementary matching all charges present in West Nile antibodies. We then used TEOS as the backbone monomer.

A total of 303 neutral, 42 positive, 28 negative and 39 (I, L, V) hydrophobic amino acids were counted for WNA. We fixed the monomer ratios at a 1:1 ratio for positive and negatively charged amino acids. We then determined the hydrophobic amino acids to monomer ratio at an experimentally determined ratio of 2:1, as not all hydrophobic amino acids are exposed in WNA. Afterward, we fixed the neutral amino acids to TEOS ratio at 1:1.77; this was determined by comparing the molecular weights of the neutral amino acids in the template against the hydrolyzed TEOS.

Synthesis of imprinted particles was a four-step procedure, the order of reagents was imperative. Briefly, for every 146 µl of TEOS: 9 µl of APS, 40 µl of cAPS and 16 µl of OTS were used. First, 15 µl of ink were mixed with 63 µl of ethanol and 10 µl of 3% NH4OH in a centrifuge tube labeled vial 2. Then, active monomers were mixed in a centrifuge tube labeled AM, in the quantities referred above. In a new centrifuge tube, labeled vial 1, 146 µl of HEPES solution, 146 µl of TEOS and all contents in centrifuge tube AM were mixed. Contents were pipette stirred, followed by addition of all contents in vial 2. Solution’s pH was then measured and adjusted by adding HCl or NH4OH as necessary to adjust the solution to a pH of 7.4 ± 0.2. After neutralizing the pH of vial 1, pH electrode was immediately cleaned and washed in DI water.

Afterward, 441 µl of purified WNA were added at a concentration of 200 µg/ml as the template. The solution was pipette mixed and left overnight to complete the reaction. Non imprinted particles (NIPs) were synthesized under identical conditions for the exception of replacing 441 µl of WNA with HEPES buffer instead. Finally, all particles were left overnight in a rotisserie at room temperature.

After synthesis, MIPs were washed by centrifuging particles at 3200 g for 5 min and discarding the supernatant, followed by addition of 1.2 ml of elution buffer to MIPs. Particles were resuspended in a vortex mixer. If large particles were noted, these were disrupted by sonicating for 1 min in a Branson 2510 sonicator. Afterward, particles were centrifuged, and washing was repeated in triplicate. Any remaining template was removed by suspending the particles in 1.2 ml of a 50% v/v mixture of glacial acetic acid and methanol for 1 h. Particles were then centrifuged, and the supernatant was discarded. Particles were then washed in 1X PBS in triplicate and resuspended in a final volume of 400 μl, no trace of protein could be detected in the supernatant using spectrophotometry with a Micro BCA kit. Particles were then stored as is at room temperature for later use. After particle washing, a total of 7 ± 2.45 mg of microparticles were collected per batch.

### Particle characterization

For particle characterization, 10 μl of MIPs were pipetted directly to a 12 mm carbon conductive tab. Particles were then lightly coated with 80:20 gold/palladium target in a sputter coater. Images were obtained using a Hitachi S-4800 SEM. Zeta potential was calculated with a ZS90 zetasizer, where a 50 μl sample was suspended in 1X PBS and diluted as needed by the equipment in a folded capillary cell (DTS1070), determined by total particle count. A total of 6 MIPs, produced in different batches, were compared.

### MIP binding characteristics

A LabAlliance Series 3 pump, a Rheodyne injector model 9725i, a GE Tricorn 10/50 column, a LabAlliance UV-VIS model 500 detector with a rise time set to 0.3 s and an SRI model 333 integrator for data collection was used for HPLC studies. MIPs were loaded to empty HPLC column to evaluate their performance. MIPs were compared against non-imprinted particles as controls.

A total of 2 MIP and 2 NIP columns were produced by transferring particles, synthesized as above, to an empty GE Tricorn 10/50 HPLC column. Particles were then immobilized in the column with silica gel by adding 500 µl of TEOS, followed by 100 µl of APS, 25 µl of cAPS, 200 µl of ink and 700 µl of ethanol; template was not used with the silica gel. Afterward, air was intentionally added by rough mixing the solution for a minute with a pipette. In approximately 45 min, solutions gelled in the HPLC columns encapsulating the particles. Finally, column packing was achieved by flowing 2x PBS at a maximum flow rate of 1 ml/min and a maximum column back pressure of 150 psi. If the back pressure was exceeded, the column was repacked. Column flow rates varied from 0.6 ml/min to 1 ml/min.

Packed columns were then equilibrated with 2x PBS, as the mobile phase, until the absorbance at 280 nm stabilized. After equilibration, 60 μl of WNA serum was loaded to a 20 μl loop in the sample injector. The sample was then injected at a flow rate of 0.5 ml/min in 2x PBS mobile phase. The first elution peak was recorded and discarded, as it constituted rejected protein from the column. After column equilibration, the mobile phase was changed to elution buffer to release retained antibody from the column. Peaks were then recorded and collected for later analysis with western blot. The collected samples were then desalted with 1x PBS buffer in a Hitrap desalting column, to prevent protein denaturation. Samples were then compared to WNA serum and WN affinity purified antibody.

Western blot was carried with a standard SDS-page acrylamide gel. The following volumes were added per well: 10 μl of molecular weight marker in well one, 5 μl of WNA serum in well two, 20 μl of affinity purified WNA in well three and 40 μl of sample collected from pH control column in well 4. Volumes were estimated based on protein concentration of collected samples. After blotting, the gel was transferred to nitrocellulose paper and developed with fluorescently labeled mice and rabbit anti-antibodies.

We used confocal imaging to determine if MIPs specifically absorbed WNA against controls. MIPs were loaded with fluorescently labeled WNA. As controls, MIPs were loaded with either fluorescently labeled BSA or BGG. As negative controls, NIPs were incubated with fluorescently labeled WNA, BSA, or BGG. All tests were then repeated a total of 4 times. The study was carried with 30 μl of pH matched MIPs or NIPs pipetted to a 0.6 ml centrifuge tube and 20 μl of the appropriate fluorescently labeled protein. Samples were then gently mixed with a pipette and vials were left overnight in a rotisserie to reach equilibrium. Samples were then centrifuged, the supernatant discarded and particles were washed twice with 300 μl of 0.03 M NaCl. Particles were then centrifuged and resuspended in 100 μl of 0.03 M NaCl. Finally, 5 μl of each sample was loaded to a microscope glass slide and viewed under a confocal microscope.

Confocal and standard bright field images were obtained in an inverted Nikon Ti-U microscope, Nikon C1 confocal system, equipped with NIS elements and EZ-C1 software. The images were analyzed using Fiji imaging software [28]. For bright field images, all particles were counted using the particle analysis tool. For confocal images, a histogram was generated and all pixels above the signal noise were counted. The pixels of the confocal images were then multiplied by their signal intensity, added together, and divided by total black pixels obtained from their respective bright field image; the average image fluorescence intensity (AIFI) was thus obtained.

We used UV-VIS spectrophotometry to determine the binding isotherm and loading capacity of MIPs. Vials were loaded with 315 μg of MIPs and 240 μl of 0.03 M NaCl solution. Afterward, nominal amounts of antibody were added per vial as follows: 1.2, 2.4, 3.6, 4.8, 6.1, 12.6, 19, 25.2, 30, 48, 70 and 95 μg of antibody per mg of MIP. Antibody concentrations were determined by Micro BCA. Each sample was conducted in quadruplets and left overnight in a rotisserie to reach equilibrium. The process was repeated with NIPs as well.

After equilibration, particles were then centrifuged at 3200 g for 5 min, the supernatant collected and a micro BCA assay conducted. Particularly, centrifugation could not precipitate all the particles from the supernatant. Thus, the absorbance of the supernatant, containing MIPs without antibody, was obtained after centrifugation; a total of 6 vials were used. Afterward, the baseline was subtracted from the absorbance values of all vials. The free ligand concentration was calculated as the difference between the protein concentration in the supernatant after equilibrium and the loaded protein before equilibrium, normalized by the solution’s volume. The bound ligand concentration was then determined by the difference of the total antibody mass added to the particles minus the free ligand mass, normalized by the mass of particles.

### Particle immunoassay study

To determine if MIPs could be used as a fluorescent immunoassay, particles were fluorescently labeled and used in a high bind 96 well plate previously loaded with antigen. MIPs were prepared as described, with the addition of 50 µl green UV dye to vial 2. A high protein binding 96 well plate was loaded with 10 µg of purified WNA in 100 µl of HEPES buffer in 9 wells. Afterward, 10 µg of BSA was added to 100 µl of HEPES buffer in 9 wells. The process was repeated with 10 µg of BGG in 9 wells. The well plate was then left overnight in an incubator at 37°C.

Afterward, a total of three solutions were made: the first solution was made with HEPES buffer, the second with 0.01% Tween 20 in HEPES buffer, and the third was with 0.005% BSA in HEPES buffer. The solutions were then loaded with 200 µl of MIPs each. Solutions were left incubating for 1 hour in a rotisserie. Meanwhile, the 96 well plate was washed with 200 µl of 0.01 % (w/v) Tween 20 in HEPES buffer a total of 3 times, per well.

After particle incubation, MIPs in standard buffer solution were added to three wells containing BSA, three wells containing BGG, and three wells containing purified WNA. The process was repeated for MIPs in BSA and Tween solutions. The well plate was then incubated again for two hours and washed five times with 0.01% (w/v) Tween 20 in HEPES. Finally, the well plate was viewed in an Olympus IX71 inverted microscope with a 4X objective, a UV exciter filter, and a green barrier filter block. A total of 27 images were obtained, particles were counted using color threshold and analyze particle filters.

## Results and Discussion

**Figure 1:**
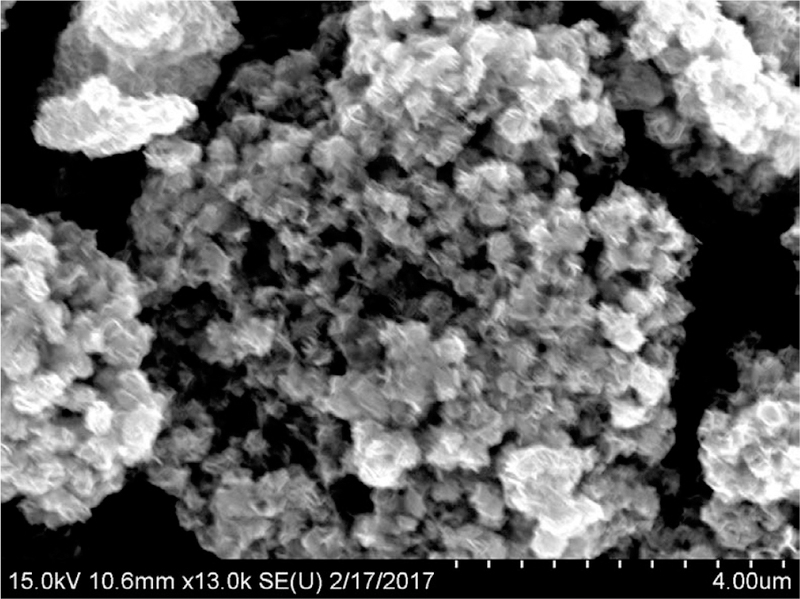
13,000x SEM image of MIP particles.

Because of the unideal synthesis conditions, we obtained silica particles with a wide size distribution (Figure 1). Where the small particles tend to form bigger aggregates and the collection of aggregates formed µm-sized particles, which are visible to the naked eye. The broad size distribution prevented the use of light scattering techniques for determining average particles size. Nonetheless, zeta potentials could be obtained, where MIPs exhibited broad potential peaks with an average negative potential of −19.5 mV with an SD of 28.7. Due to the large zeta potential of the particles, these will aggregate easily. Thus, the majority of particles will precipitate from the solution after a given amount time.

### MIP binding characteristics

**Figure 2:**
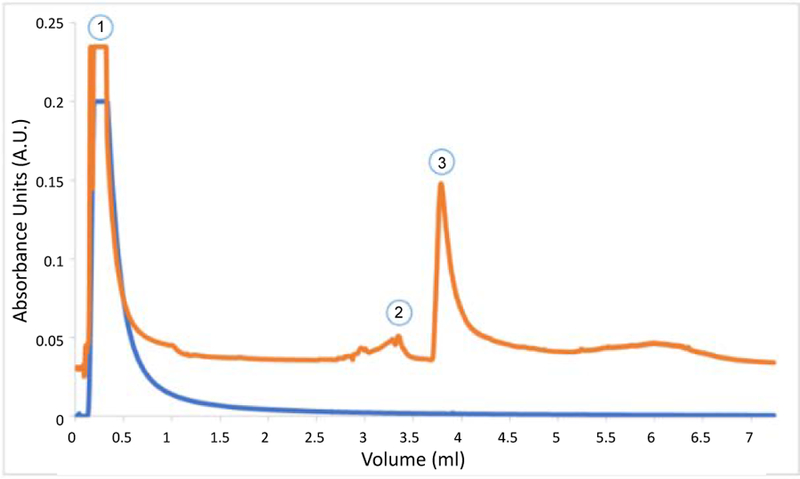
MIP binding traces. The top trace is the elution from a West Nile antibody MIP column. The bottom trace shows the elution from a non-imprinted control column. Peak 1 is the protein rejected by the columns. Peak 2 on top trace is attributed to the phase change as no protein was detected with a micro BCA assay. Peak 3 is retained protein by the MIP column.

**Figure 3:**
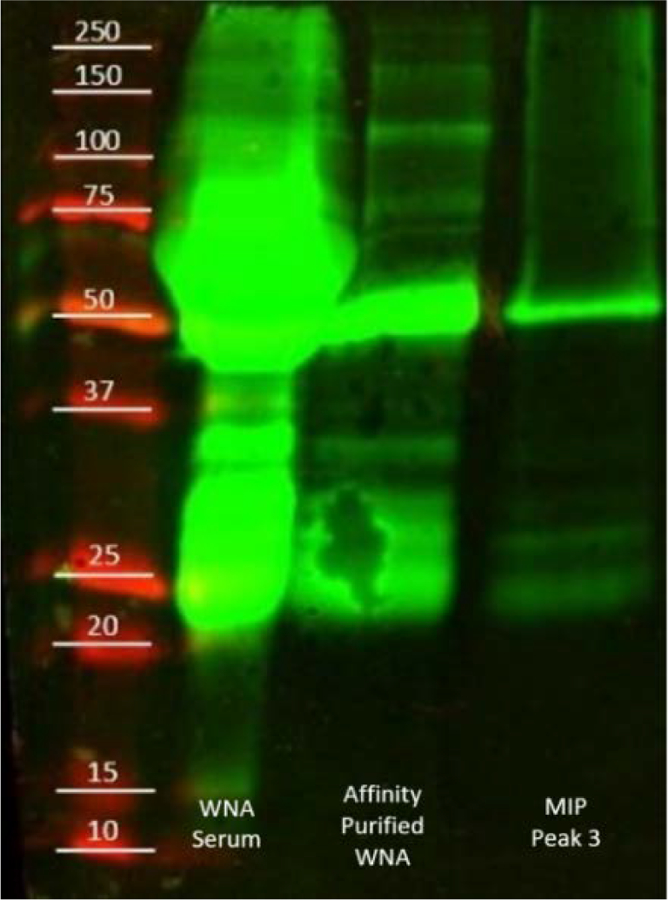
Western blot of the elution from MIP column. Well 1: molecular weight marker; Well 2: WNA serum; Well 3: Affinity purified WNA; Well 4: sample from MIP column.

The HPLC offset comparison (Figure 2), shows traces obtained from a MIP and a NIP column. The initial peak, rejected protein, was present in both columns, while the MIP column contained a small peak at an elution volume of 3.00 ml. The elution contained no measurable protein by micro BCA. Therefore, the peak was attributed to the change of mobile phase to elution buffer, and the sample was discarded. Afterward, MIP column showed a peak at an elution volume of 3.75 ml. The collected protein aliquot was compared against WN serum and purified WNA by western blot (Figure 3). The purified antibody presented bands at 50 and 25 kDa, which corresponds to the heavy and light chain of the antibody. The elutions from the MIP column exhibited the common bands at 50 kDa and 25 kDa. Thus, MIPs successfully captured WNA.

**Figure 4:**
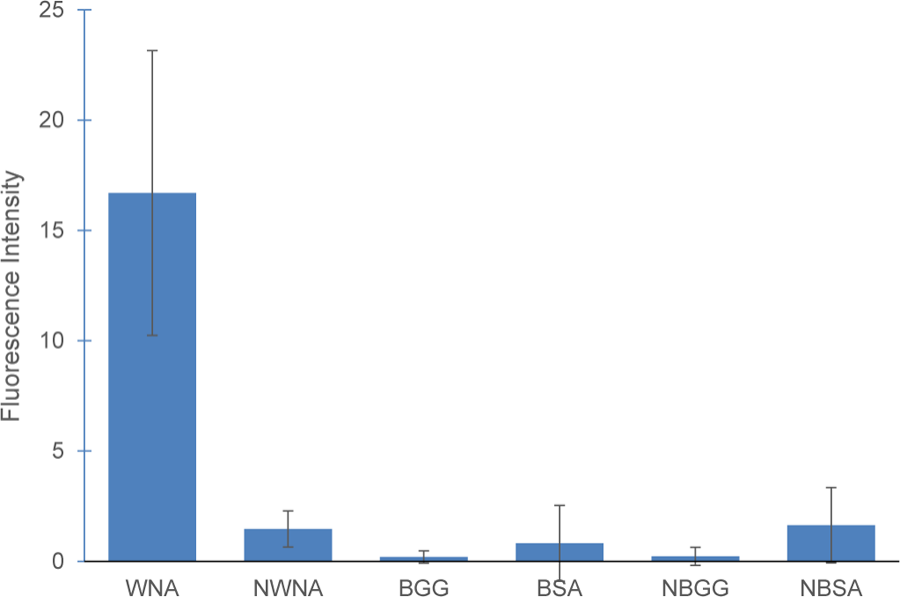
Fluorescent protein absorption in particles. Where WNA is WNA absorbed in MIP particles. NAWN are WNA absorbed by NIPs. BGG and BSA are controls absorbed by MIP particles. NBGG and NBSA are controls absorbed by NIP particles. Each column represents the average of 4 images obtained per group.

After determining that MIPs specifically bound to WNA, the confocal study was carried. WNA, BSA and BGG were fluorescently labeled using ATTO 495 NHS ester. After incubating MIPs with their respective proteins, a total of 24 images were analyzed and their AIFI were plotted (Figure 4). MIPs had a preferential binding of West Nile antibodies as indicated by a significantly higher fluorescence in the images obtained. MIPs incubated with WNA had an AIFI 16 times greater than images obtained with NIPs incubated with WNA, commonly referred to as the imprinting factor. While the AIFI of MIPs with WNA was 27 times greater than any AIFI of MIPs incubated with BSA or BGG, known as the selectivity factor. The AIFI of MIPs in the presence of BSA or BGG was similar to all NIP samples with overlapping SD values; thus, there was no statistical difference between any of the controls. These factors compare favourably with literature where imprinting factors of 4–8 and selectivity coefficients of 5–7 have been reported [29–31]. All p-values were below 0.0015 when comparing MIPs to NIPs or any of its controls.

**Figure 5:**
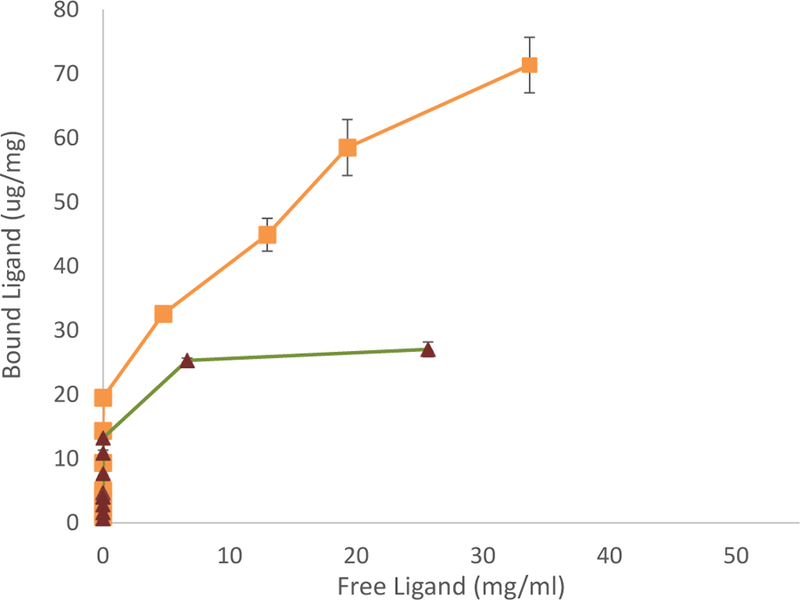
Binding isotherms for West Nile MIPs, and their NIPs. Squares represent MIPs equilibrated in WNA and Triangles NIPs equilibrated in WNA.

For the binding isotherms, a total of 48 samples were obtained; the results are shown in Figure 5. Particularly, NIPs showed an approximate loading capacity of 28 μg/mg of WNA. Since the controls in the confocal study had no statistical difference between NIPs, BSA, and BGG, only NIPs were used. Meanwhile, MIPs adsorbed approximately 80 μg of WNA per mg of MIPs. Thus, MIPs bound almost triple WNA than NIPs in equilibrium studies and without sample washing. The p-value at the loading limit was 0.000003. Finally, the dissociation constant of MIPs was calculated at Kd=57.45 μM. While the dissociation constant provides with an insight to MIP-antigen interactions, its value is difficult to compare with antibodies, partly due to the large loading capacity of MIPs and their wide size distribution.

### Fluorescently labeled MIPs as immunoassay

**Figure 6:**
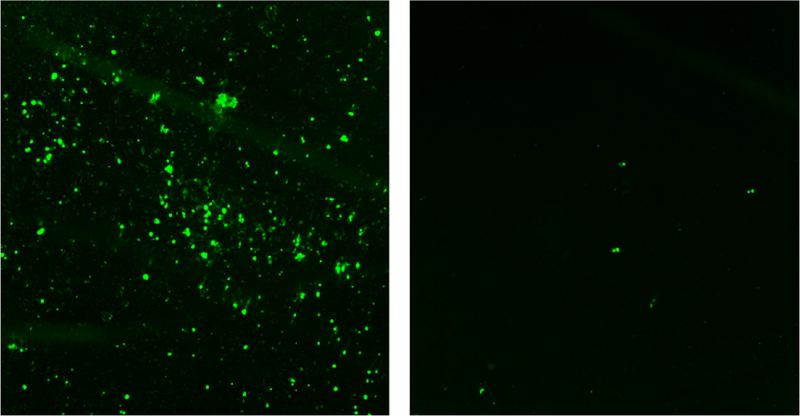
Fluorescently labelled immunoassay with MIPs. Left: positive WNA sample; Right: negative BSA sample.

**Figure 7:**
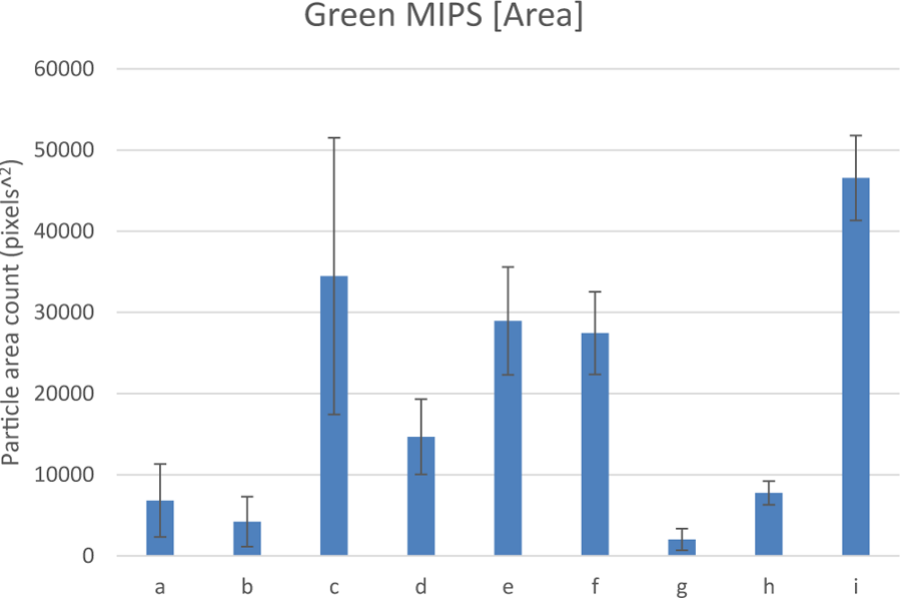
Fluorescently labeled MIPs immunoassay, total particles area count. Where wells a-c had unblocked MIPs in wells loaded with a) BSA b) BGG and c) WNA. While wells d-f were presented with MIPs blocked with tween 20 in wells loaded with d) BSA e) BGG and f) WNA. Wells g-I had MIPs blocked with BSA in wells loaded with g) BSA h) BGG and i) WNA.

The objective of the test was to determine if fluorescent MIPs could be used as an ELISA assay. A 96 high protein binding well plate, previously incubated with positive and negative samples, was used to immobilize MIPs. The test was carried with MIPs in a non-blocking buffer, a hydrophilic blocker buffer with Tween 20, and a hydrophobic blocker buffer with BSA. A comparison of fluorescent images is shown in Figure 6. The images show a high number of fluorescent particles binding to a positive well sample, whereas only a few particles are bound to a negative control well. The results are summarized in Figure 7, where MIPs show a statistically significant higher number of particles in WNA wells.

Interestingly, blocking MIPs with tween 20 dramatically increased background signal, essentially eliminating the recognition capabilities of MIPs. Tween 20 blocked particles had a p-value of 0.38 of WNA against negative controls. BSA blocked MIPs had no significant difference over unblocked MIPs in average total particle counts, with a p-value of 0.33. However, BSA blocked particles had narrower deviation standards, where BSA blocked MIPs had a p-value of 0.0019 against negative controls, while unblocked MIPs had a higher p-value of 0.05.

## Conclusion

Molecular imprinting of West Nile antibody was achieved by using a silica sol-gel method, where active monomers were fixed at a 1 to 1 ratio. The Hydrophobic monomer was experimentally determined at a 2 to 1 ratio. Finally, TEOS was established at a 1 to 1.77 ratio. Monomer volumes were calculated from the template’s crystal structure, based on amino acid charges and their counts. By systematically determining monomer volumes, MIP formulation can be easily updated to other proteins by downloading crystallographic data of the desired template. Particularly, WNA MIPs bound specifically to its template with a loading capacity of 80 μg/mg, while non-specific protein loading capacity was 28 μg/mg. After triplicate washing, MIPs retained 27 times more WNA than BSA or BGG in confocal images.

Fluorescently labeled MIPs demonstrated their potential as immunoassays, where BSA blocked MIPs can be used instead of primary and secondary antibodies. The identification of a positive test was as simple as looking under a microscope. MIPs behaved reliably and the differences between positive and negative results were evident. Because fluorescent MIPs work similarly as an ELISA test, they can be utilized in low resource labs that are already equipped for ELISA tests, with the addition of a fluorescent microscope. Finally, as the imprinting process improves, particles could be used with standard microscopy and even lateral flow assays, thus facilitating the development of affordable and robust immunoassays in the future.
